# Floating-Harbor Syndrome Treated With Recombinant Human Growth Hormone: A Case Report and Literature Review

**DOI:** 10.3389/fped.2021.747353

**Published:** 2021-11-05

**Authors:** Hui Bo, Lihong Jiang, Jiaqi Zheng, Jie Sun

**Affiliations:** ^1^Jinghai Clinical College of Tianjin Medical University, Tianjin, China; ^2^Department of Pediatrics, Jinghai District Hospital, Tianjin, China; ^3^Department of Pediatrics, General Hospital of Tianjin Medical University, Tianjin, China

**Keywords:** short stature, Floating-Harbor syndrome, *SRCAP* gene, recombinant human growth hormone, treatment

## Abstract

**Introduction:** We aimed to summarize the clinical characteristics of Floating-Harbor syndrome (FHS) and the effect of recombinant human growth hormone (rhGH) to increase height.

**Methods:** The clinical manifestations, gene sequencing results, treatment, and regression of one child with FHS were reported at the Department of Pediatrics, General Hospital of Tianjin Medical University, in July 2020. PubMed was searched using the keyword “Floating-Harbor Syndrome” up to March 2021 to obtain clinical information on children with FHS for review.

**Results:** The child, who was a male aged 6 years and 9 months, presented to the clinic with main complaints of delayed language development since childhood and a short stature for 4 years. The child's short stature, peculiar facial features, delayed language development, and delayed bone development were considered alongside genetic testing and Sanger sequencing to verify the results. A heterozygous mutation (c.7401delC; p.Ile2468Phefs^*^7) was identified in exon 34 of the *SRCAP* gene, which was a frameshift mutation, and Sanger verification showed that neither parent had this mutation. The child was administered subcutaneous injection of rhGH (0.13 U/kg/day) and was followed up regularly. At the time of writing, the child had been treated for 6 months and was 7 years and 3 months old with a height of 106.3 cm (−3.69 SDS), which was a height increase of 6.3 cm. The patient did not complain of discomfort during treatment and presented normal laboratory tests results. Twenty-two children with FHS treated with rhGH were included in the literature review, and most of these patients demonstrated an increase in height SDS without adverse effects.

**Conclusion:** Short stature, delayed skeletal maturation, impaired language expression, intellectual deficits, and peculiar facial features are the main clinical features of FHS. rhGH can be used as a treatment to increase height in patients with FHS, but its effectiveness and safety still need to be monitored in larger sample sizes over longer periods of time.

## Introduction

Floating-Harbor syndrome (FHS) is a rare autosomal dominant disorder. The main clinical features of FHS are short stature, peculiar facial features, and delayed speech development (1). Typical characteristic facial features include a triangular face, deep-set eyes, long eyelashes, low-set ears, a wide nasal bridge, a short philtrum, a wide mouth, and thin lips. FHS is caused by heterozygous mutations in the SNF2-related cAMP response element-binding protein (CREBBP) activator protein (*SRCAP*) gene (OMIM: 611421). Most mutations are spontaneous mutations; however, there are occasional reports of autosomal dominant familial inheritance (2). In this paper, we retrospectively analyzed the clinical data of a child with FHS who was treated with recombinant human growth hormone (rhGH) for a short stature. We also reviewed the relevant literature to provide a theoretical basis for clinical management of FHS.

## Case Description

**Figure 1:**
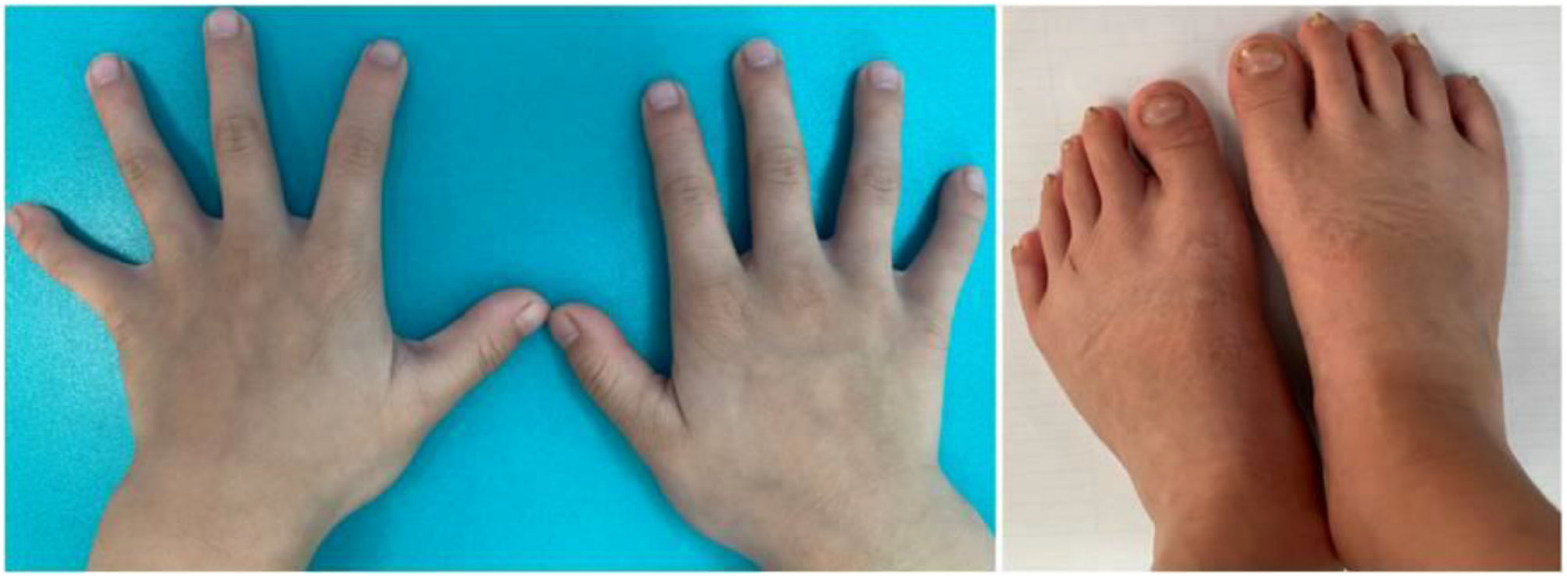
Photographs of the child's small hands and feet, thin and short fingers, fifth-finger clinodactyly, and hypoplastic nails on the feet.

**Figure 2:**
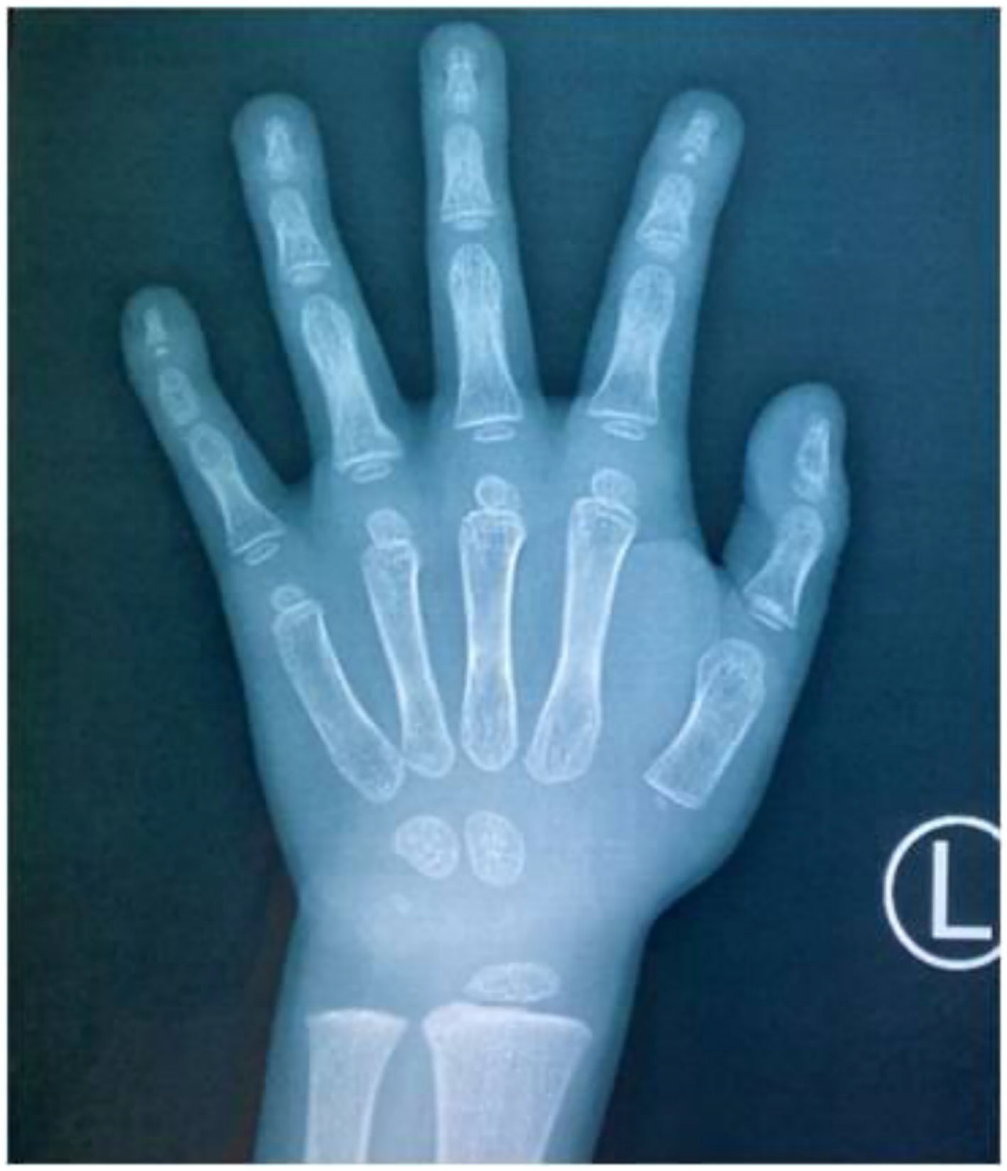
Direct radiography of the child's left hand. The bone age was 4 years (3 years behind the patient's actual age), the finger bones were short and thin, the distal phalanges were prominent, the little finger was inwardly curved, and the middle phalanges of the little finger were irregularly shaped.

The child, who was a male aged 6 years and 9 months, was admitted to the pediatric clinic of the General Hospital of Tianjin Medical University in July 2020 with main complaints of delayed speech development and a short stature for 4 years since childhood. The child was born from a gravida 2 para 2 mother, was born at full term, and had no history of birth injury or asphyxia. The child's birth length was 50 cm (50th percentile), and his birth weight was 3.25 kg. The child could verbalize “mom” at 4 years of age, and he started language training at 5 years of age. The child's mother was 167 cm tall, his father was 165 cm tall, and his brother was 9 years old with normal growth and intellectual development. The parents were not consanguineous and denied any family history of hereditary disease. Physical examination results were as follows: height, 100 cm [−4.50 standard deviation score (SDS)]; weight, 15 kg (−3.78 SDS); finger spacing, 99.4 cm; sitting height, 53 cm; sitting height/lower body length, 1.13 (P3 to P50); head circumference, 49 cm; blood pressure, 90/50 mmHg. The child was well-proportioned, had peculiar facial features (a triangular face, deep-set eyes, a wide nasal bridge, thin lips, a small lower jaw, sparse teeth, and multiple dental caries), small hands and feet, thin and short fingers, fifth-finger clinodactyly, hypoplastic toenails (shown in Figure 1 for the hands), and delayed speech development. The child had a screaming voice when crying and laughing. Testicular volume was ~1 ml, and penis length was ~3 cm [<-2 standard deviations (SDs)]. An auxiliary examination showed that routine bloods, electrolytes, liver, and kidney function, and thyroid function were normal. The chromosome number was 46, with the XY genotype. Insulin-like growth factor-1 (IGF-1) concentration was 95.9 ng/ml (−1 SD to M) and insulin-like growth factor-binding protein-3 concentration was 3.54 μg/ml (mean +1 SD). The concentration of 25-hydroxyvitamin D was 34.74 nmol/L, suggesting vitamin D deficiency. Abdominal and adrenal ultrasound showed no abnormalities. Echocardiography showed a widened ascending aorta, left ventricular enlargement, aortic valve thickening and stenosis (mild), and regurgitation (near-moderate). Testicular ultrasound showed left testicular dimensions of 1.5 × 1.0 × 0.6 cm, a left testicular volume of 0.64 ml (<-2 SD), right testicular dimensions of 1.5 × 0.9 × 0.7 cm, and a right testicular volume of 0.67 ml (<-2 SD). Direct radiography of the left hand showed a bone age of 4 years, which was 3 years behind the patient's actual age. The phalanges were thin and short, which was especially prominent in the distal phalanges. The little finger was inwardly curved, and its middle phalanges had an irregular morphology (Figure 2). Direct radiography showed no bone abnormalities and no visible caudal bone, which suggested delayed bone development. Pituitary magnetic resonance imaging showed a normal pituitary morphology, with a height of ~3.8 mm and no abnormal signals, and the pituitary stalk was centered with no thickening.

The child had peculiar facial features, a short stature, delayed speech development, and significantly delayed bone development. The child was on high clinical alert for FHS and underwent an improved genetic examination. After medical ethical review and receipt of informed consent from the child's family, whole-exome testing and Sanger sequencing were completed and verified. The results showed that a heterozygous mutation (c.7401delC; p.Ile2468Phefs^*^7) was present in exon 34 of the *SRCAP* gene, which is a frameshift mutation that is not reported in the Human Gene Mutation Database. Using Mutation Taster software to predict protein function, American College of Medical Genetics and Genomics guidelines determined that the probably pathogenic variant, PVS1 + PM2, which occurs at a low frequency in the normal population, is pathogenic. The mutation, which is highly conserved across species, substitutes the encoded isoleucine for phenylalanine, causing an early termination signal at downstream amino acid 7, producing a truncated protein and resulting in loss of function. Sanger verification showed that neither parent had this variant.

The child's clinical characteristics and genetic test results led to a clear diagnosis of FHS. The patient's parents strongly desired to increase the child's height. After reviewing the relevant literature, there were no contraindications to rhGH treatment for FHS. Therefore, the child was treated with rhGH [0.13 IU/(kg-d)] subcutaneously and vitamin D (800 IU/d), and was followed up regularly. At the time of writing, the child had been treated for 6 months and was 106.3 cm tall (−3.69 SDS) at the age of 7 years and 3 months, with a height increase of 6.3 cm. During the treatment period, the child's routine bloods, liver and kidney function, electrolytes, thyroid function, and fasting glucose insulin were normal. 25-Hydroxyvitamin D was increased compared with the pre-treatment concentration, IGF-1 fluctuated between −1 SD and +1 SD, and scoliosis was not present. The collective results are shown in Table 1.

**Table 1:** Follow-up data of the patient who was treated with rhGH, including age, height, differential, scoliosis, blood count, liver and kidney function, and electrolyte concentrations.

**Age**	**Height (cm)**	**HtSDS**	**BA (yrs.) (difference between BA and CA)**	**Scoliosis**	**Routine bloods**	**Liver and kidney function**	**Electrolytes**	**IGF-1 (ng/ml)**	**IGFBP3 (μg/ml)**	**FT3 (pmol/L)**	**FT4 (pmol/L)**	**TSH (uIU/ml)**	**GLU (mmol/T)**	**INS (mU/L)**	**25(OH)D (nmol/L)**
6 yrs. 9 mo.	100	−4.5	3.8(−2.9)	N	N	N	N	95 (−0.91 SD)	3.54 (+0.16 SD)	5.29	12.56	1.7	3.97	1	34.74
7 yrs.	103.5	–	–	–	N	–	–	160 (−0.14 SD)	4.39 (+0.80 SD)	5.25	14.48	1.81	4.62	5.8	59.29
7 yrs. 3 mo.	106.3	−3.69	–	N	N	N	N	257 (+0.67 SD)	–	5.5	19.82	2.6	–	–	–

*yrs, years; mo, months; N, normal; –, not measured; HtSDS, height standard deviation score; BA, bone age; CA, chronological age; FT3, free triiodothyonine; TSH, thyroid-stimulating hormone; GIU, blood glucose; INS, insulin; 25(OH)D, 25-hydroxyvitamin D*.

### Literature Review

The keyword “Floating-Harbor Syndrome” was used to search PubMed up to March 2021 to obtain clinical data on children with FHS. There are more than 100 reported cases of FHS worldwide, and a short stature is one of the common features of patients with FHS; however, only a few children with FHS have been treated with rhGH. We searched PubMed for all studies describing rhGH treatment in children with FHS up to March 2021, with the following criteria: (i) a clinical or genetic diagnosis of FHS; (ii) application of rhGH treatment; and (iii) inclusion of detailed information about the growth of the child and response to treatment. Twenty-one children with FHS who met the inclusion criteria were retrieved (2–11), and a total of 22 cases, including the present case, were identified. Table 2 summarizes the basic information of children with FHS who were treated with rhGH. The age at onset and the duration of treatment varied in these children. Most children responded well to treatment with an increased growth rate and an increase in height SDS compared with before treatment, and no adverse effects were observed. Three children exhibited a poor response to rhGH treatment (2, 7) which was possibly related to inappropriate acceleration of bone age during rhGH treatment. In addition, some children developed precocious puberty (10) and were treated with gonadotropin-releasing hormone analog combination therapy.

**Table 2:** Basic information of 22 patients with FHS treated with rhGH.

**Literature**	**ID**	**Sex**	***SRCAP*** **mutation site**	**Growth hormone deficiency**	**Age at the start of rhGH treatment (yrs.)**	**Height SDS at the start of rhGH treatment**	**Duration of rhGH treatment (yrs.)**	**Height SDS at the time of final evaluation**	**ΔHtSDS**
Zhang et al. (2)	1	Male	c.7330C>T (p.Arg2444Ter)	Yes	5.2	−4.5	1	−4.4	0.1
	2	Male	c.7245_7246delAT	No	4.8	−4.1	4.1	−2.5	1.6
	3	Female	c.7330C>T (p.Arg2444Ter)	No	5	−3.8	1.6	−3.4	0.3
	4	Male	c.7330C>T (p.Arg2444Ter)	No	6.5	−5.3	4.3	−3.8	1.5
	5	Male	c.7303C>T (p.Arg2435Ter)	No	5.1	−4.5	4.3	−3.3	1.2
	6	Female	c.7303C>T (p.Arg2435Ter)	No	1.5	−4.2	2.2	−2.9	1.3
	7	Female	c.7466C>G (p.Ser2489Ter)	No	2.3	−4.1	0.3	−3.8	0.3
Garcia et al. (11)	8	Female	–	No	3.5	−3.1	5	−2.4	0.7
Nagasaki et al. (7)	9	Male	c.7330C>T (p.R2444x)	No	10	−4.9	2	−3.6	1.3
Galli-Tsinopoulou et al. (3)	10	Female	–	Yes	5.4	−3.8	2	−2.2	1.6
Hiyun et al.	11	Female	c.7330C> T (p.Arg2444*)	No	8	−3.3	4.5	−2.7	0.6
Homma et al. (4)	12	Male	c.7330C> T (p.Arg2444*)	No	4.9	−3.1	8.1	−1.1	2
	13	Female	c.7303C>T (p.Arg2435*)	No	4.2	−3.4	2.8	−2.6	0.8
	14	Male	c.7262dupG (p.Arg2421fs)	No	7.9	−3	2.5	−2	1
	15	Female	c.7330C>T (p.Arg2444X)	No	10.4	−2.1	4.1	−2.5	−0.4
Seifert et al. (6)	16	Female	c.7395delA (p.Val2466Tyrfs*9)	No	2	−2.5	3	−2	0.5
	17	Female	c.7218dupT (p.GIn2407Serfs*36)	No	2	−3.2	9	−1.8	1.4
	18	Female	c.6985C>T (p.Arg2329*)	No	5.3	−3.4	–	−1.7	1.7
Cannavò et al. (8)	19	Female	–	Yes	9.1	−2.9	1.5	−1.9	1
Stagi et al. (10)	20	Female	–	No	10.1	−2.2	–	−1.2	1
Wieczorek et al. (9)	21	Female	–	No	5.3	−3	3.6	−0.9	2.1
Present case	22	Male	c.7401delC (p.lle2468Phefs*7)	No	6.8	−4.5	0.5	−3.6	0.9

*ΔHtSDS height SDS at final assessment – height SDS at start of treatment (column head section)*.

## Discussion

FHS is a rare autosomal dominant disorder that occurs as a consequence of heterozygous mutations in the *SRCAP* gene. The syndrome was named at the hospital where the first two cases were described (12, 13). Such mutations are mostly spontaneous, but there have been occasional reports of autosomal dominant familial inheritance. Nonsense or frameshift mutations in the terminal portion of the *SRCAP* gene in exon 34 (exon 33 in two cases in the literature) may result in formation of carbon terminally truncated variants of SRCAP proteins with missing functional domains. The dominant-negative effect of the truncated protein variant encoded by the *SRCAP* gene is FHS (14). Currently, more than 40 pathogenic variants of the *SRCAP* gene associated with FHS have been reported (mainly nonsense or frameshift mutations in exon 34), and most of these mutations occur between codons 2407 and 2517, while only two variants are nonsense mutations in exon 33. c.7330C>T (p.Arg2444Ter) is the most common variant, while c.7303C>T (p.Arg2435Ter) is the second most common variant (2). The heterozygous mutation (c.7401delC; p.Ile2468Phefs^*^7) in the *SRCAP* gene of our patient was a *de novo* frameshift mutation in exon 34, with the mutation site located between codons 2407 and 2517, which is consistent with the previous description. In addition, this mutation was a *de novo* mutation, expanding the mutation spectrum of FHS.

The *SRCAP* gene is located on chromosome 16p11.2, which contains a 40,989-bp coding region and 34 exons encoding a SNF2-related chromatin remodeling ATPase (the SRCAP protein), which is abundant in the nuclei of human cells (2). The SRCAP protein plays a crucial role in essential cellular pathways, such as chromatin remodeling, gene expression, DNA damage response, and cell division (4). The SRCAP protein includes several discrete functional domains: the SNF2-like ATPase structural domain, the centrally located CREBBP-binding domain, the N-terminal HSA structural domain, and three C-terminal AT-hook structural domains. The SRCAP protein can exert transcriptional regulation by activating CREBBP, which activates a number of transcription factors and mediates gene expression. Thus, in addition to its role in chromatin remodeling, SRCAP affects a variety of signaling pathways, such as the Notch signaling pathway and steroid receptor-mediated transcriptional pathways. Mutations in the gene encoding CREBBP can lead to Rubinstein-Taybi syndrome (RTS), so there is some overlap in the clinical manifestations of FHS and RTS. In addition to the CREBBP-binding domain, SRCAP also carries other structural domains located at the C-terminus (2316–2971), which activate transcription independently of CREBBP. These structural domains contain portions of the AT-hook motif. The AT-hook is a small DNA-binding motif consisting of ~9 amino acids found in many DNA-binding proteins. These proteins play a key role in chromatin organization and in the expression of genes that control essential cellular processes (4). A nonsense or frameshift mutation in exon 34 or 33 of the *SRCAP* gene produces a truncated protein that results in loss of all AT-hook structural domains at the C-terminus, and SRCAP proteins that have lost the AT-hook structural domain cannot activate the *CREBBP* gene efficiently, resulting in stunted development and FHS. The multiple roles of SRCAP can explain the phenotypic variation in FHS, which is characterized not only by facial features, growth disturbances, and mild skeletal malformations, but also by gastrointestinal, cardiac, and genitourinary disorders (15).

The main features of children with FHS are a short stature, special facial features, delayed bone age, and delayed speech development. Facial features include a triangular face, deep-set eyes, long eyelashes, a high nasal bridge, a short philtrum, a low-hanging nasal columella, a wide mouth, a thin upper lip, and low-set ears. Skeletal abnormalities include wide thumbs, short fingers, and tapered epiphyses [other manifestations, such as pseudarthrosis-like clavicle deformity, unilateral or bilateral clavicle deformity, and hip dysplasia, have also been reported (15)]. A high-pitched voice and nasal tone and delayed speech development are common in patients with FHS and are characterized by delayed expressive language. Children with FHS have varying degrees of intellectual impairment, and some patients also have psycho-behavioral abnormalities, such as attention deficit hyperactivity disorder, obsessive-compulsive disorder, and anxiety disorder. There are also clinical manifestations that cannot be considered as distinctive features of FHS and that thus require comprehensive screening in children with FHS. This is because certain abnormalities affect the method of clinical management, such as oto-ocular abnormalities (hyperopia, strabismus, nystagmus, conductive hearing loss); ear, nose, and throat problems (cleft lip, pseudolabial, palatopharyngeal atresia, posterior nostril atresia); dental abnormalities (dental caries, widely spaced, and small teeth); congenital heart disease; gastrointestinal disorders (gastroesophageal reflux, celiac disease, constipation); and genitourinary abnormalities [renal stones, hydronephrosis, renal cysts, hypouric acidemia, cryptorchidism, small penis, small testes (16–18)]. We report the present case of a child with typical facial features, a short stature, delayed speech development, and delayed bone age, consistent with the reported clinical manifestations of FHS.

A short stature is the most characteristic feature of FHS. The exact mechanism by which *SRCAP* mutations cause a short stature has not been fully elucidated. Some studies suggest that the skeletal growth plate is responsible for bone elongation and height. Impaired linear growth (i.e., height) must be due to primary or secondary disorders of growth plate chondrocytes caused by dysfunction of the skeletal growth plate (19). In the growth plate, chondrocytes proliferate, hypertrophy, and secrete cartilage extracellular matrix components. This process generates new cartilage tissue, which is subsequently remodeled into bone tissue. Eventually, new bone is gradually formed in the growth plate, thus allowing bones to become longer and children to grow taller. This process involves multiple signaling pathways, including the Indian hedgehog (IHH) protein, parathyroid-related protein (PTHrP), fibroblast growth factor, and bone morphogenetic protein (BMP). The Notch signaling pathway is downstream of the IHH, BMP, and PTHrP pathways and inhibits chondrocyte differentiation (20). Therefore, abnormalities in the Notch pathway lead to dysregulation of chondrocyte proliferation and maturation, resulting in delayed long bone development, which supports the notion that direct effects on growing cartilage may affect the growth phenotype of patients with FHS, which is consistent with the findings of Nagasaki et al. (7). In addition, abnormalities in these signaling pathways may lead to skeletal deformities, such as short fingers. The child in the present case also had mild skeletal deformities (thin and short fingers, fifth-finger clinodactyly, hypoplastic nails on the foot, and short middle phalanges of the fifth metacarpal). Children with FHS usually have a severe delay in bone age until 6 years of age, and bone age may be delayed or normal in patients between 6 and 12 years of age and then significantly accelerated. Accelerated senescence of growing cartilage suggests that a delayed bone age does not translate into room for height growth and reflects abnormal chondrocyte development.

In addition, it has been suggested in the literature that a short stature in children with FHS is associated with growth hormone deficiency (3), growth hormone neurosecretory dysfunction (8), and IGF-1 signaling defects (11). Only a few of the more than 100 cases of FHS have been reported to have growth hormone deficiency. Garcia et al. (11) suggested that FHS may lead to impaired IGF-1 signaling and affect height because of the difference between the growth response to rhGH and serum IGF-1 (at the upper limit of normal levels during treatment). Nowadays, an increasing number of studies show that rhGH treatment can increase growth rate and improve the height of children with dwarfism. rhGH treatment maintains IGF-1 at high levels, thus having a stimulatory effect on proliferation and hypertrophy of growth plate chondrocytes, which in many cases non-specifically accelerates linear growth, thus partially compensating for the deficiency in some unrelated molecules affecting the growth plate (19). The child reported in the present case had a height increase of 6.3 cm after 6 months of rhGH treatment, with IGF-1 fluctuations between −1 SD and +1 SD and an increase in height SDS, but the treatment period was short, and the effect of late treatment needs to be closely monitored.

In addition to the case described in this study, 21 other children have been reported in the literature, most of whom showed accelerated growth and improved height SDS. During rhGH treatment, the IGF-1 concentration remained normal or at the upper limit of the normal range, indicating reasonable sensitivity to rhGH. According to the data, the effects of rhGH treatment were different. Although they had the same genotype, they may have had a different phenotype. Alternatively, it might be related to a longer duration of rhGH application and reduced sensitivity to growth hormone. However, some studies have suggested that the mean adult height of children treated with rhGH was significantly greater compared with that of patients who did not receive treatment, suggesting that rhGH treatment might be a good option to improve linear growth in children with FHS (4).

A report in the literature (21) described a possible association between rhGH therapy in patients with FHS and spinal cord embolism. This discovery suggested that periodic evaluation of patients with FHS undergoing growth hormone treatment should include regular lower limb neurological examinations. Another report (22) revealed that a child developed nephrotic syndrome during growth hormone therapy; thus, growth hormone was discontinued. One paper (23) reported that four cases of FHS developed significant cerebrovascular disease, including three cases of hypertension; therefore, it is recommended that children with FHS should undergo blood pressure monitoring annually from adolescence, regular renal ultrasound in adulthood, and timely screening for neurological disease. In the present study, however, no side effects were reported in 22 children treated with rhGH. We agree that children with FHS should be routinely monitored for growth, bone age, and secondary sexual characteristics. Patients with FHS should also undergo annual ophthalmologic evaluation, hearing screening, evaluation of dental problems, blood pressure monitoring, renal function tests, and urinary ultrasound.

In summary, the clinical presentation of FHS is complex, with special facial features, short stature, and delayed language expression being the main features of the disease. Timely and accurate diagnosis is beneficial for early intervention. We reported a male patient aged 6 years and 9 months with a clinical presentation and genetic diagnosis of FHS. The patient's height SDS increased after 6 months of rhGH treatment. The mechanism of growth disorders in children with FHS is still unclear, and the effectiveness and safety of rhGH treatment still needs to be monitored in larger sample sizes over longer periods of time.

## Data Availability Statement

The original contributions presented in the study are included in the article/supplementary material, further inquiries can be directed to the corresponding author/s.

## Ethics Statement

Written informed consent was obtained from the individual(s), and minor(s)' legal guardian/next of kin, for the publication of any potentially identifiable images or data included in this article.

## Author Contributions

LJ contributed to the conception and design of the study. JZ performed the statistical analysis. HB wrote the first draft of the manuscript. JS wrote sections of the manuscript. All authors contributed to manuscript revision, read, and approved the final version for submission.

## Conflict of Interest

The authors declare that the research was conducted in the absence of any commercial or financial relationships that could be construed as a potential conflict of interest.

## Publisher's Note

All claims expressed in this article are solely those of the authors and do not necessarily represent those of their affiliated organizations, or those of the publisher, the editors and the reviewers. Any product that may be evaluated in this article, or claim that may be made by its manufacturer, is not guaranteed or endorsed by the publisher.
